# Intraoperative echocardiographic delineation of the high take-off coronary ostia during an extensive surgical repair of the bicuspid aortic valve and dilated sinotubular junction: a case report

**DOI:** 10.1186/s12871-016-0250-x

**Published:** 2016-10-04

**Authors:** Hyun Ju Jung, Won-Kyoung Kwon, Sung Jun Lee, Nazri Mohamed, Bo-Mi Shin, Jinyoung Lee, Tae-Yop Kim

**Affiliations:** 1 Department of Anesthesiology, Uijeongbu St. Mary Hospital, The Catholic University of Korea, Uijeongbu, Gyounggi-do Korea; 2 Department of Anesthesiology, Konkuk University Medical Center, Konkuk University School of Medicine, 120-1 Neungdong-ro, Hwayang-dong, Gwangjin-gu, Seoul Korea; 3 Department of Chest Surgery, Konkuk University Medical Center, 120-1 Neungdong-ro, Hwayang-dong, Gwangjin-gu, Seoul Korea; 4 Cardiothoracic Anesthesiology and Perfusion Unit, Hospital Tengku Ampuan Afzan, 25100, Jalan Tanah Putih, Kuantan, Pahang Malaysia; 5 Department of Anesthesiology, Konkuk University Medical Center, 120-1 Neungdong-ro, Hwayang-dong, Gwangjin-gu, Seoul Korea; 6 Department of Business, Sungkyunkwan University, 25-2 Sungkyunkwan-ro Jongro-gu, Seoul, Korea; 7 Department of Anesthesiology, Konkuk University Medical Center, Konkuk University School of Medicine, 120-1 Neungdong-ro, Gwangjin-gu, Seoul 143-729 Republic of Korea

**Keywords:** Aortic valve repair, Coronary artery, Coronary ostia, Transesophageal echocardiography

## Abstract

**Background:**

This study reports the efficacy of intraoperative transesophageal echocardiography (TEE) for evaluation of high take-off coronary ostia and proximal coronary arterial flows as an alternative to preoperative coronary angiography.

**Case presentation:**

In a 65-year old male undergoing the bicuspid aortic valve (BAV) repair and the extensive remodeling of dilated sinus and tubular junction, and preoperative coronary angiography were unsuccessfully completed due to an allergic reaction to the contrast medium. Intraoperative TEE by employing various 3-dimensional volume images of coronary ostia and Doppler tracings of the coronary arterial flows enabled a thorough pre-procedural evaluation of the high take-off coronary arteries and post-procedural evaluation by confirming the absence of any compromise in coronary arterial flow.

**Conclusion:**

In the present case, intraoperative application of various TEE imaging modalities enabled comprehensive evaluation of high-taking off coronary artery, as an alternative to preoperative coronary angiography, in a patient undergoing an extensive aortic valve and aortic root repair procedure.

**Electronic supplementary material:**

The online version of this article (doi:10.1186/s12871-016-0250-x) contains supplementary material, which is available to authorized users.

## Background

Application of various sutures and fabric materials is an essential part of aortic valve (AV) repair with remodeling of aortic root [1, 2]. Considering the potential to injure the adjacent structures, such as coronary ostia or proximal coronary arteries, it requires thorough evaluations of coronary arteries before the extensive AV repair procedure. However, coronary angiography, which has been used as a gold standard for evaluating coronary arterial pathologies, can be infeasible or contraindicated due to various reasons. A previous literature showed use of intraoperative 3-dimensional (3D) transesophageal echocardiography (TEE) enabled delineation of coronary artery fistulae and suggested possible efficacy of intraoperative 3D-TEE for pre- and post-procedural evaluation of the proximal coronary arterial flow, as an alternative method to coronary angiography [3].

In the present case of bicuspid AV repair with extensive remodeling of the dilated aortic root, intraoperative TEE was employed for pre- and post-procedural evaluations of the high-take off coronary arteries.

## Case presentation

A 55-year-old male underwent an AV repair procedure for stenotic bicuspid AV and remodeling of dilated sinotubular junction and ascending aorta. Preoperative two-dimensional (2D) transthoracic echocardiography (TTE) showed severe AR through bicuspid AV and dilated sinus valsalva, sinotubular junction and ascending aorta. High-taking off coronary arteries were also noted on the TTE. However, preoperative coronary angiogram was not successful due to sudden drop of blood pressure and symptoms suggesting his allergic reaction to the iodide-containing contrast medium at the beginning of coronary angiography. Without performing any further preoperative evaluation for the coronary artery, the surgical plan for BAV repair was established. Other surgical plans regarding the coronary pathology were supposed to be modified upon the results of intraoperative pre-procedural TEE examination.

**Fig. 1:**
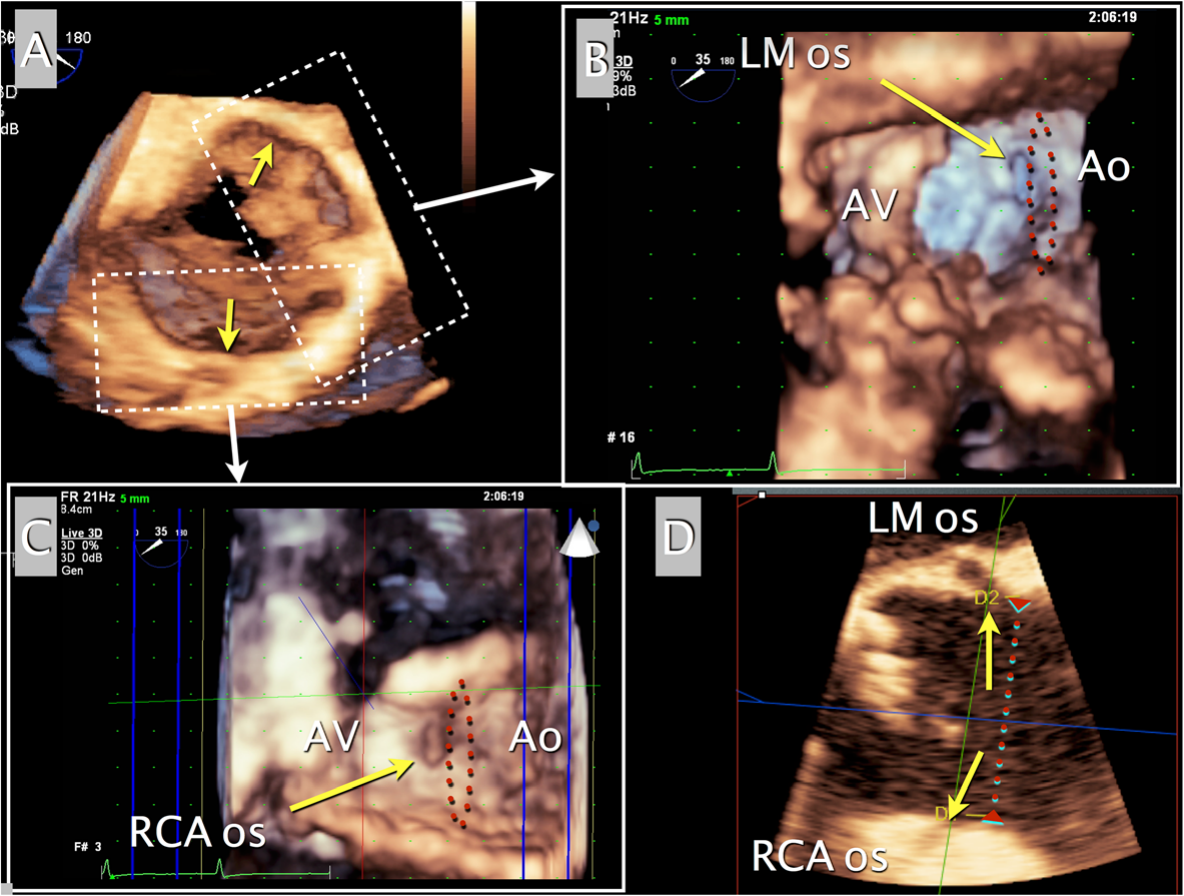
Intraoperative pre-procedural TEE imaging. A real-time 3D Zoom “en face” image of the AV, seen from an aortic perspective, confirmed a bicuspid AV (**a**). Rendered and rotated enface images with built-in 3D-grids (dot-to-dot distances of 2 and 5 mm) focused on the left main and right coronary ostia (*yellow arrows*) revealed their high take-off with distances less than 3 mm from the STJ (red dot circles) (**b** and **c**). Additional multi-planar cut (tomographic) images with a different axis, rendered from the 3D dataset of the AV using 3DQ software in QLAB™ (Philips Healthcare, Bothell, WA), confirmed the short distance of 2 ~ 3 mm from the STJ to the RCA and left main ostium (**d**). Ao, aorta; AV, aortic valve; LA, left atrium; LM os, left main coronary ostium; LV, left ventricle; RA, right atrium; RCA os, right coronary ostium

Intraoperative 2D and 3D TEE (iE33™ and X7-2 t™, Philips Healthcare, Bothell, WA, USA) confirmed the thickened and calcified BAV and dilated and ellipsoidal aortic root. 2D midesophageal (ME) AV short- and long-axis (SAX and LAX) images delineated the thickened bicuspid AV with the annular diameters of 19 mm. Sinus valsalva, sinotubular junction and ascending aorta were all dilated with diameters of 29 mm, 26 mm and 32 mm, respectively. Their 2D TEE with color Doppler images demonstrated severe AR through the entire valvular coaption. The right coronary artery (RCA) taking-off from sinus adjacent to sinotubular junction was noted in ME AV LAX image. A live 3D-zoom volume image of the AV, which was acquired and rotated to generate an “en face” AV image seen from the ascending aortic perspective, showed a stenotic bicuspid AV with both commissural fusions at the 5 and 11 o’clock positions (Fig. 1-a). Additional 3D “en face” images derived from 3D-zoom images focused on aortic annulus and both coronary ostia showed much greater annular diameter (21 mm vs. 19 mm in 2D TEE) and the left main (LM) and highly taking-off right coronary (RCA) ostia arising at the 1 and 6 o’clock positions of the enlarged sinus valsalva (diameter 32-33 mm), respectively (Video 1). Applying built-in 3D-grids (dot-to-dot distance was 2 or 5 mm) on the “en face” images also provided a rough estimation of the distances between the coronary ostia, the sinotubular junction and AV cuspal attachment (Fig. 1-b and c). A multi-planar 2D-cut image rendered from the 3D volume dataset also enabled precise measurements of the distances from the coronary ostia to the sinotubular junction, which were all <3 mm (Fig. 1-d).

**Fig. 2:**
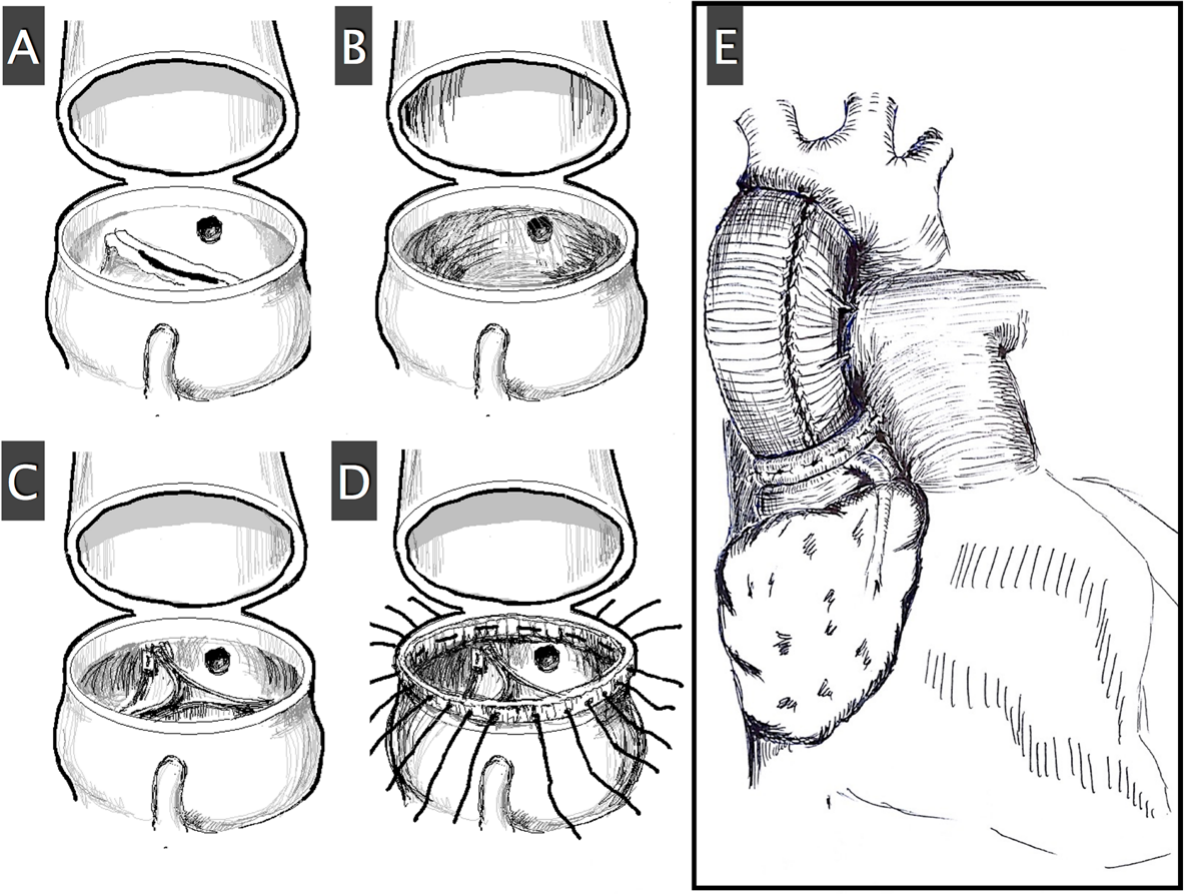
Schematic of the AV repair and aortic root remodeling procedure. A horizontal aortotomy was made 10 mm above both coronary ostia (**a**). The AV cusp structures were excised fully through the aortotomy (**b**). A tricuspidized AV was reconstructed using the leaflet extension technique and three ready-to-use templates of bovine pericardium patches, and coaptation sutures were placed at the new commissural ends (**c**). Non-expandable inner and outer rings were applied at the new sinotubular junction (STJ) with penetrating sutures to reduce the dilated STJ, and a synthetic graft was subsequently wrapped around the dilated ascending aorta for further reinforcement after the aortotomy was closed (**d** and **e**)

Extensive AV repair surgery was performed through a sternotomy under moderate hypothermic cardiopulmonary bypass (CPB) with cold cardioplegia administered antegrade and retrograde (Fig. 2) [1, 2]. Horizontal aortotomy was made at the position 10 mm higher than usual, considering highly taking-off coronary ostia. The AV cuspal structures were excised entirely through the aortotomy, and one of the 2 commissures in the bicuspid AV became a base for creating 3 new commissures that had the same intercommissural distances. Then, three ready-to-use templates of bovine pericardium patches were sutured to the cuspal suture lines which had been placed so as not to injure the coronary ostia. The sinotubular junction was remodeled to match its diameter to annular diameter by making penetrating interrupted sutures through the non-expandable inner-and outer-rings (fabric strips) at the new sinotubular junction. The lowest margin of the inner ring was placed carefully so as not to occlude the coronary ostia. Then, the aortotomy was closed, and the ascending aorta was externally wrapped with a synthetic graft for reducing the diameter of the new sinotubular junction to match that of aortic annulus and further external reinforcement [2].

**Fig. 3:**
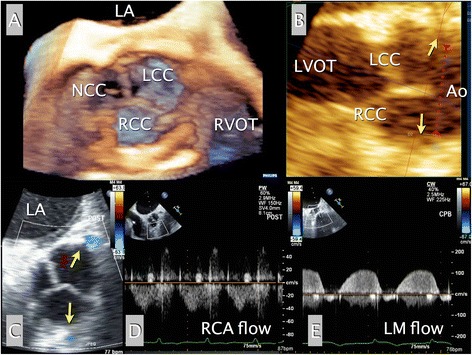
Intraoperative post-procedural TEE imaging. Real time 3D TEE “en face” image of the repaired AV view, seen from the aortic perspective, revealed the tricuspidized AV (**a**). Additional multi-planar cut (tomographic) images with different axes, rendered from 3D datasets of the AV, revealed preservation of both coronary ostia just below the sinotubular junction (**b**). Midesophageal AV short-axis image with color Doppler showed patency of the LM and the proximal RCA (*arrows*) (**c**). Pulsed-wave Doppler tracing demonstrated an intact flow pattern of the RCA (**d**) and the LM (**e**). Ao, aorta; AV, aortic valve; LA, left atrium; LM, left main coronary artery; LCC, left coronary cusp; LV, left ventricle; LVOT, LV outflow tract; NCC, non-coronary cusp; RA, right atrium; RCA, right coronary artery; RCC, right coronary cusp

Immediately after CPB-weaning, post-repair TEE revealed a successful AV tricuspidization with sufficient valve opening (>3.6 cm^2^), which resembled a normal tricuspid AV (Fig. 3-a, Video 2). No residual AR was noted. Additional multi-planar tomographic images, rendered from the 3D volume dataset, revealed intact coronary ostia (Fig. 3-b).

2D color Doppler and pulsed-wave Doppler tracing of the LM and the proximal RCA revealed intact systolic and diastolic coronary arterial flow patterns, confirming the absence of any compromise due to the extensive repair procedure (Fig. 3-c, d and e, Video 2).

Institutional research board of Konkuk University Medical Center approved and written informed consent was obtained from the patient for the present case report.

## Conclusions

In the present case, application of intraoperative TEE enabled thorough pre- and post-procedural evaluations for his high-take off coronary arteries in a patient who was undergoing an extensive AV repair procedure and unable to perform preoperative coronary angiographic evaluation. The intact coronary arterial flow pattern in Doppler tracings confirmed the absence of their injury or dysfunction due to the extensive repair procedure. Adding 3D-TEE on the conventional 2D and Doppler imaging could provide delineation of coronary ostia and the proximal coronary arterial flows.

Recently, magnetic resonance imaging without iodide-containing contrast has been introduced for evaluations of coronary artery for patients in whom coronary angiography is not suitable due to allergic reaction to the contrast media [4].

Meantime, the coverage and efficacy of 2D-TEE were limited in evaluating the anatomic information of coronary ostia and coronary flow due to their inherent disadvantages: the need to frequently manipulate the TEE probe and the difficulty of delineating structures located beyond its image plane. By contrast, 3D-TEE volumetric dataset could provide more intuitive and anatomically oriented tomographic or “en face” images seen from various perspectives by rendering or rotating the acquired volume image, even without manipulating the TEE probe. Multiple 2D tomographic images rendered from the 3D dataset enabled much more accurate measurements of the distances between the coronary ostia and the sinotubular junction in the present case. As a result, the horizontal aortotomy was made at a much higher location than usual, and the sinotubular junction inner ring and the new commissural lines for tricuspidizing AV were placed with sufficient distance from the opening of both coronary ostia, so as not to occlude the coronary ostia or compromise the coronary flows. 3D-TEE’s ability to provide various images seen from various perspectives may expand the efficacy of intraoperative TEE in complex cardiac procedure, such as AV repair procedure in the present case.

Of note, ability of 3D-TEE providing various tomographic images out of the single volume dataset seemed to be useful for easy, accurate and reproducible measurements of the elliptically dilated aortic root dimensions as in the present case. In AV repair procedures for dilated aortic root, accurate determination of the size of eccentrically dilated aortic annulus or Sinus Valsalva is important for selecting the type of procedures, aortic root “remodeling” or “reimplantation”, as well as for predicting the overall outcome of the procedure [5–7]. 3D-TEE is able to provide accurate aortic diameters by avoiding 2D-TEE’s underestimation, probably due to the foreshortening.

However, optimal post-repair TEE imaging of the coronary ostia was partly unsatisfactory due to acoustic shadows from the various sutures and fabric materials used for the leaflet extension and remodeling of the sinotubular junction, as in the present case. In this circumstance, the patency of the coronary ostia could be evaluated by using the color imaging as well as pulsed wave Doppler tracing of the proximal coronary arterial flows.

In conclusion, intraoperative TEE employing various TEE imaging and Doppler modalities enabled a thorough pre-procedural evaluation of high take-off coronary arteries, as an alternative to preoperative coronary angiography, and the success of the extensive AV repair in a patient undergoing complex AV repair and remodeling of dilated aortic root.



**Video 1 (additional file 1)**. A real-time 3D “en face” TEE image of the AV, seen from the aortic perspective, confirmed a bicuspid AV (A). The rendered and rotated “en face” images focused on the left main and the right coronary ostia revealed their high take-off in locations extremely close to the sinotubular junction (B and C). Ao, aorta; AV, aortic valve; LA, left atrium; LM os, left main coronary ostium; LV, left ventricle; RVOT, right ventricular outflow tract; RCA os, right coronary ostium.

**Video 2 (additional file 2)**. Real-time 3D “en face” TEE image of the repaired AV, view seen from the aortic perspective, revealed a tricuspidized AV with sufficient opening (A). Midesophageal AV short-axis image with color Doppler revealed intact coronary arterial flows of the left main and the right coronary arteries (yellow arrows) (B). Midesophageal AV long-axis image with color Doppler showed a sufficient AV opening without any residual AR and intact right coronary arterial flow (yellow arrow) (C). Ao, aorta; AV, aortic valve; LA, left atrium; LCC, left coronary cusp; LV, left ventricle; RA, right atrium; NCC, non-coronary cusp; RCC, right coronary cusp.


## Abbreviations

2D: 2-dimensional
3D: 3-dimensional
AR: Aortic regurgitation
AS: Aortic stenosis
AV: Aortic valve
CPB: Cardiopulmonary bypass
TEE: Transesophageal echocardiography

## References

[1] Song MG, Yang HS, Choi JB, Shin JK, Chee HK, Kim JS (2014). Aortic valve reconstruction with leaflet replacement and sinotubular junction fixation Aortic valve reconstruction with leaflet replacement and sinotubular junction fixation. Aortic valve reconstruction with leaflet replacement and sinotubular junction fixation: early and midterm results. Ann Thorac Surg.

[2] Song MG, Yang HS, Choi JB, Shin JK, Chee HK, Kim JS (2014). Aortic valve reconstruction with use of pericardial leaflets in adults with bicuspid aortic valve disease: early and midterm outcomes. Tex Heart Inst J.

[3] Kim J, Yu GY, Seok J, Oh CS, Kim SH, Kim TY (2014). Imaging of coronary artery fistulae by using intraoperative three-dimensional transesophageal echocardiography. Anesth Analg.

[4] Prakken NH, Cramer MJ, Olimulder MA, Agostoni P, Mali WP, Velthuis BK (2010). Screening for proximal coronary artery anomalies with 3-dimensional MR coronary angiography. Int J Cardiovasc Imaging.

[5] le Polain de Waroux JB, Pouleur AC, Pasquet A, Gerber BL, Noirhomme P (2009). Mechanisms of recurrent aortic regurgitation after aortic valve repair: Predictive value of intraoperative transesophageal echocardiography. JACC Cardiovasc Imaging.

[6] Augoustides JG, Szeto WY, Bavaria JE (2010). Advances in aortic valve repair: Focus on functional approach, clinical outcomes, and central role of echocardiography. J Cardiothorac Vasc Anesth.

[7] Tian D, Rahnavardi M, Yan TD (2013). Aortic valve sparing operations in aortic root aneurysms: remodeling or reimplantation?. Ann Cardiothorac Surg.

